# Functional Mobility and Fall Risk Assessment in Lower Limb Amputees: Insights From the Timed Up and Go Test

**DOI:** 10.7759/cureus.89297

**Published:** 2025-08-03

**Authors:** João Santos-Faria, Flávio Ribeiro, Vitor Sousa, Joana Santos-Costa, João Branco

**Affiliations:** 1 Physical Medicine and Rehabilitation, Unidade Local de Saúde de Coimbra, Coimbra, PRT; 2 Physical Medicine and Rehabilitation, Hospital Senhora da Oliveira, Guimarães, Guimarães, PRT

**Keywords:** fall risk, functional mobility, lower limb amputation, prosthetic rehabilitation, timed up and go test

## Abstract

Background: Lower limb amputation (LLA) significantly affects mobility and increases fall risk, particularly in individuals with higher-level amputations and lower functional capacity. Effective tools to assess fall risk are essential in rehabilitation.

Objective: This study was conducted at the Department of Physical Medicine and Rehabilitation, Coimbra University Hospital (HUC), Coimbra, Portugal. This study aimed to evaluate the performance of the Timed Up and Go (TUG) test in individuals with unilateral LLA, compare TUG times by amputation level (transfemoral vs. transtibial) and functional level (K2 vs. K3), and determine the predictive value of TUG for fall risk.

Methods: A retrospective observational study was conducted with 99 unilateral LLA patients using prostheses. Participants were assessed using the TUG test, and data on demographics, functional classification, and fall history were collected. Statistical comparisons and ROC curve analysis were performed to identify significant differences and the optimal TUG cutoff for fall risk prediction.

Results: Transfemoral amputees had significantly slower TUG times than transtibial amputees (17 vs. 12 seconds; p < 0.001). Similarly, K2 amputees in both groups had significantly longer TUG times compared to their K3 counterparts (TF: 30 seconds vs. 14 seconds; TT: 27 seconds vs. 12 seconds; p < 0.001). Individuals with a history of falls also had significantly higher TUG times (26 vs. 10 seconds; p < 0.001). A TUG threshold of 18 seconds demonstrated a sensitivity of 100% and specificity of 69% (AUC = 0.81) for fall prediction.

Conclusion: The TUG test is a reliable and practical tool for assessing functional mobility and fall risk in individuals with unilateral LLA. Performance varies significantly with amputation level and functional classification, and a cutoff of 18 seconds effectively identifies individuals at increased risk of falling.

## Introduction

Lower limb amputation (LLA) is a life-altering condition that significantly impairs mobility, independence, and quality of life [1]. Even with advances in surgical techniques and prosthetic technology, individuals with LLA face persistent challenges in gait efficiency, postural control, and energy expenditure [2]. Among these, increased risk of falling is one of the most serious concerns, as it is associated with further injury, functional decline, and social withdrawal [3].

Falls in amputee populations are alarmingly frequent. Studies estimate that more than 50% of individuals with LLA fall at least once annually, and many experience multiple falls [4,5]. Factors contributing to this risk include decreased proprioception, asymmetric gait patterns, reduced muscle strength, and limitations in balance [6]. Understanding and mitigating fall risk in this population is therefore a priority in rehabilitation medicine.

Functional mobility assessment tools are critical in identifying individuals at risk and tailoring rehabilitation programs. One such tool is the Timed Up and Go (TUG) test, a simple and reliable method that measures the time taken for a person to rise from a chair, walk three meters, turn, return, and sit down [6]. Originally developed for geriatric patients, the TUG test has shown strong reliability and validity across multiple clinical populations, including individuals with neurological and orthopedic conditions [7].

In amputee populations, the TUG test has gained interest as a potential proxy for gait stability and fall risk. Previous research has linked TUG performance with K-level classification, energy cost, and balance control [8,9]. However, its specific role in stratifying risk by amputation level (transtibial (TT) vs. transfemoral (TF)) and functional classification remains underexplored.

A study by Engenheiro et al. [5] demonstrated that transfemoral amputees have significantly worse functional outcomes than transtibial amputees, due in part to higher biomechanical demands and energy costs. Similarly, research by Vllasolli et al. [10] confirmed the biomechanical disadvantage faced by TF amputees, leading to lower functional scores.

This study aims to evaluate TUG performance in individuals with unilateral LLA, compare results between TF and TT amputees and between K2 and K3 functional levels, and assess the ability of the TUG test to predict fall risk. By identifying clinically relevant thresholds, this study seeks to enhance the utility of TUG in everyday clinical practice.

## Materials and methods

A retrospective observational study was conducted involving adult individuals with unilateral LLAs. The study population included both transfemoral and transtibial amputees who had been using prosthetic devices for more than two years and demonstrated ambulatory ability. Participants were consecutively enrolled between March 2020 and March 2023 from the amputee outpatient clinic of Department of Physical Medicine and Rehabilitation, Coimbra University Hospital (HUC), a central hospital in Coimbra, Portugal.

Inclusion criteria were adults aged 18 years or older, unilateral LLA (transfemoral or transtibial), prosthetic use for more than two years, and most recent prosthetic fitting at least one year prior to the study. All participants were ambulatory, with or without assistive devices. Exclusion criteria included bilateral amputations, cognitive impairment, non-cooperation during assessments, prostheses requiring revision or repair, and functional level K0 (no potential for ambulation), according to the Medicare Functional Classification Levels.

Comprehensive demographic and clinical data were collected, including age, sex, amputation level, cause of amputation, prosthetic use, and K-level classification. Relevant comorbidities such as diabetes mellitus, hypertension, dyslipidemia, chronic kidney disease (CKD), and cardiovascular disease were also documented.

All included participants had already completed their prosthetic rehabilitation process and were using optimized prosthetic components, including knee joints, selected and adjusted by a multidisciplinary rehabilitation team based on individual functional needs and gait assessment.

Functional mobility was assessed using the TUG test, administered in a standardized manner by the same trained observer. All assessments were performed in a controlled clinical environment to ensure consistency and minimize external variability.

This study involved a retrospective analysis of anonymized clinical data collected during routine care. In accordance with Portuguese legislation, no formal ethics approval was required. All procedures complied with the ethical standards of the Helsinki Declaration.

Statistical analysis

All statistical analyses were performed using IBM SPSS Statistics for Windows, Version 25.0 (IBM Corp., Armonk, NY). Descriptive statistics summarized demographic and clinical characteristics, with continuous variables presented as means or medians, depending on the data distribution. Categorical variables were expressed as frequencies and percentages.

The normality of continuous variables was assessed using the Shapiro-Wilk test. For normally distributed data, parametric tests such as the independent samples t-test were employed to compare means between groups. For non-normally distributed data, non-parametric tests were used. Associations between categorical variables were analyzed using the chi-square test or Fisher’s exact test, as appropriate. The relationship between TUG performance and fall risk was further explored using receiver operating characteristic (ROC) curve analysis. The area under the curve (AUC) was calculated to evaluate the discriminative ability of the TUG test, and an optimal cutoff value for predicting fall risk was determined based on sensitivity and specificity. A p-value of <0.05 was considered statistically significant for all analyses.

## Results

A total of 99 unilateral lower limb amputees were included in the study, comprising 46 transfemoral and 53 transtibial amputees. No significant differences were observed between the two groups regarding demographic and clinical characteristics, including age, sex, K-level, cause of amputation, time since amputation, and the prevalence of comorbidities such as diabetes, hypertension (HTN), dyslipidemia, CKD, and cardiovascular disease (Table 1).

**Table 1:** Demographic and clinical characteristics of participants Values are presented as mean, median, or number (percentage), as appropriate. The p-values refer to comparisons between transfemoral and transtibial amputees and were calculated using chi-square or Fisher’s exact test for categorical variables, and independent-samples t-test or Mann-Whitney U test for continuous variables, depending on data distribution. Statistical significance was set at p < 0.05.

	Transfemoral (n = 46)	Transtibial (n = 53)			p-value
Demographic and clinical characteristics					
Age (mean; years)	63.1	62.3			0.404
Sex, N (%) masculine	36 (78.3)	42 (79.2)	X^2^ = 0.014		0.905
Time since amputation (median; years)	22.5	20.0			0.413
Active labor N (%) yes	13 (28.3)	12 (22.6)	X^2^ = 0.412		0.543
Comorbidities					
Hypertension N (%) yes	33 (71.7)	36 (67.9)	X^2^ = 0.169		0.680
Dyslipidemia N (%) yes	28 (60.8)	30 (56.6)	X^2^ = 0.185		0.667
CKD N (%) yes	6 (9.6)	4 (7.5)	Fisher's exact test		0.507
Cardiovascular disease N (%) yes	9 (19.6)	10 (18.8)	X^2^ = 0.007		0.930
Cause of amputation					
Vascular(N)	12	22	X^2^ = 7.484		0.112
Traumatic (N)	29	29			
Neoplastic (N)	4	0			
Infeccious (N)	1	1			
Congenital (N)	0	1			
Medicare Functional Classification Level					
K1 (N)	1	0	X^2^ = 3.392		0.335
K2 (N)	19	16			
K3 (N)	26	36			
K4 (N)	0	1			

Transfemoral amputees exhibited significantly longer TUG times compared to transtibial amputees (17 seconds vs. 12 seconds; p < 0.001). Stratification by K-level revealed that K2 amputees in both groups had significantly longer TUG times compared to their K3 counterparts (TF: 30 seconds vs. 14 seconds; TT: 27 seconds vs. 12 seconds; p < 0.001) (Table 2). 

**Table 2:** Timed Up and Go (TUG) times by K-level and amputation type. Results in seconds (s). * Denotes a statistically significant p-value (p < 0.05). All p-values were derived using independent-samples t-tests.

	K2	K3	p-alue
Transfemoral (s)	30	14	p < 0.05*
Transtibial (s)	27	12	p < 0.05*

Eight falls were recorded during the study period, involving five TF and three TT amputees (Table 3).

**Table 3:** Fall incidence in transfemoral and transtibial amputees

	Yes	No	Total
Transfemoral (n)	5	41	46
Transtibial (n)	3	50	53
Total	8	91	99

Participants who experienced falls demonstrated significantly longer TUG times than those who did not (26 seconds vs. 10 seconds; p < 0.001).

**Figure 1:**
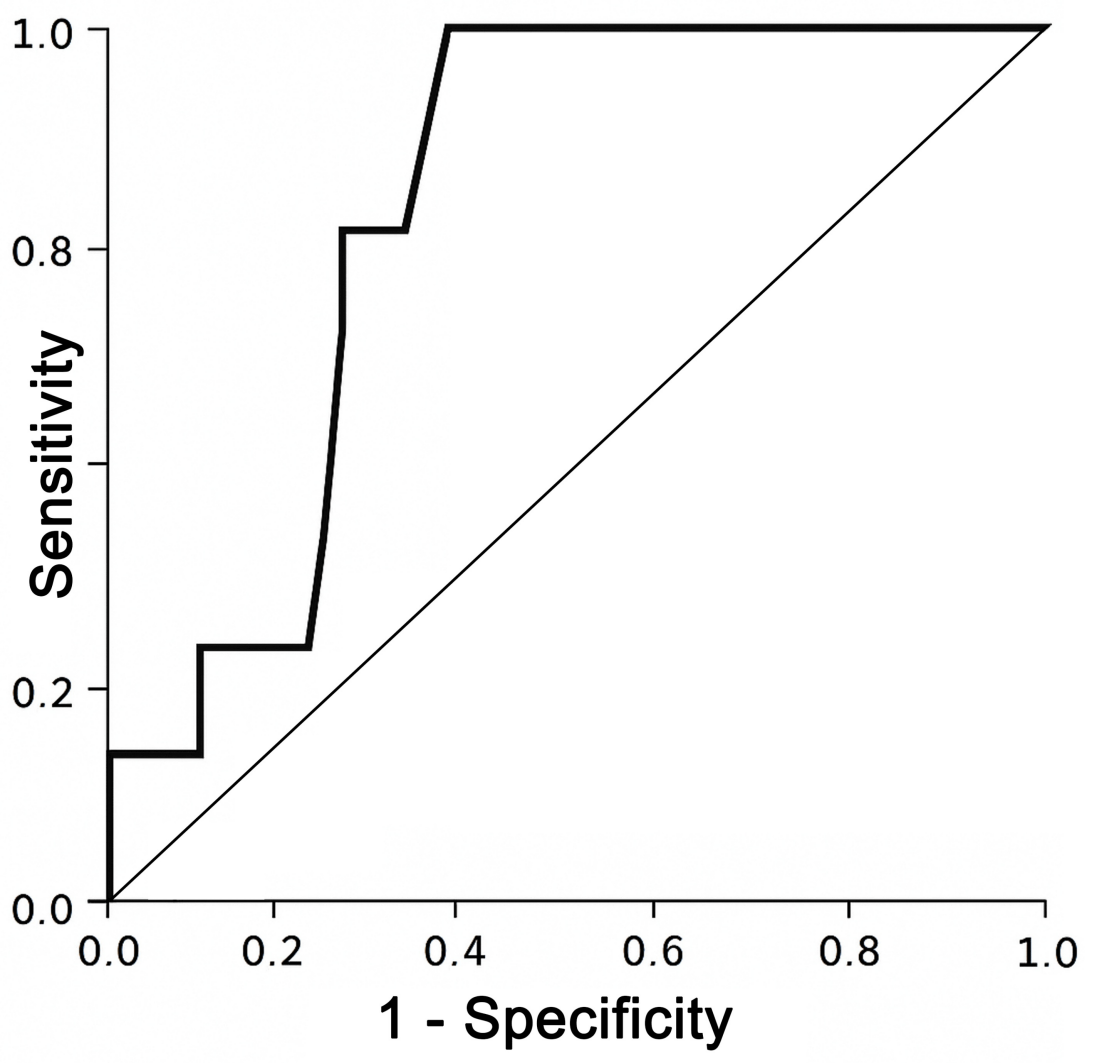
Receiver operating characteristic (ROC) curve analysis

For identifying fall risk in this population, a TUG time cutoff of 18 seconds was found to provide optimal predictive value, yielding a sensitivity of 100% and a specificity of 69%. Receiver operating characteristic (ROC) analysis showed an area under the curve of 0.81, indicating good discriminative ability (Figure 1).

## Discussion

This study provides further evidence supporting the use of the TUG test as a reliable tool for assessing functional mobility and identifying fall risk in individuals with unilateral lower limb amputation.

Our findings demonstrate that transfemoral amputees have significantly longer TUG times compared to transtibial amputees, corroborating previous studies that highlight the increased energy cost, reduced stability, and decreased gait efficiency associated with higher-level amputations [10,11]. The absence of the knee joint in TF amputees disrupts the natural gait cycle, increases prosthetic complexity, and often leads to reduced confidence during ambulation [12].

Similarly, participants classified as K2-level exhibited significantly worse TUG performance than those at K3, which aligns with the clinical expectations of K-level descriptors. The Amputee Mobility Predictor and other functional scales have shown consistent links between K-level and ambulation performance [8,9]. This reinforces the utility of TUG as a complementary measure to prosthetic functional classification.

Importantly, individuals with a history of falls had significantly slower TUG times. This observation supports existing literature that associates TUG performance with balance deficits and fall risk in both amputee and non-amputee populations [3,13,14]. One of the most compelling insights from this study is the predictive value of the TUG test for fall risk. The identified cutoff time of 18 seconds, associated with excellent sensitivity (100%) and moderate specificity (69%), provides a practical benchmark for clinicians to identify individuals at heightened risk of falls. The ROC curve analysis, with an AUC of 0.81, underscores the robustness of TUG as a discriminative tool in this context. This is consistent with findings from Dite et al. [13], who suggested that thresholds above 19 seconds may indicate recurrent fallers in this population.

It is worth noting that the fall rate observed in our sample was lower than that reported in other studies involving lower limb amputees. This may be attributed to the characteristics of our population, which included individuals who had already completed rehabilitation and were followed in a specialized outpatient clinic. All participants had stable prosthetic use for over two years, with individualized gait training and component optimization, potentially reducing fall incidence.

Although the TUG test is not a comprehensive fall-risk assessment on its own, its simplicity, cost-effectiveness, and predictive utility make it an ideal screening tool in routine rehabilitation settings. It can be administered quickly, requires no specialized equipment, and provides immediate, interpretable results [15,16]

Furthermore, our findings are consistent with data from the Portuguese study by Engenheiro et al. [5], which also highlighted the poorer functional outcomes among TF amputees and the need for targeted interventions in this subgroup.

It is worth noting that some recent literature emphasizes complementing the TUG test with dynamic balance assessments, gait symmetry analysis, or patient-reported outcome measures to capture the full complexity of post-amputation mobility and fall risk [17].

Limitations

While this study provides valuable insights, several limitations warrant consideration. The sample was drawn from a single center, potentially limiting generalizability to broader amputee populations. In addition, the study design did not account for psychosocial or environmental factors that may influence mobility and fall risk. Moreover, while comorbidities such as diabetes, HTN, dyslipidemia, CKD, and cardiovascular disease were accounted for and showed no significant group differences, their individual impact on mobility outcomes was not deeply explored. Future research should incorporate multidimensional assessments, including psychological resilience, access to advanced prosthetic technologies, and environmental barriers, to capture the complexity of real-world ambulation.

## Conclusions

The TUG test is a robust and practical measure for assessing functional mobility and fall risk in individuals with lower limb amputations. The test's simplicity, reliability, and predictive capacity make it a valuable addition to clinical practice, enabling personalized rehabilitation interventions and fall prevention strategies. Future research should aim to validate these findings in larger, more diverse populations and explore the integration of TUG with other multidimensional mobility assessments to capture the complexity of real-world ambulation.
